# The seal louse (*Echinophthirius horridus*) in the Dutch Wadden Sea: investigation of vector-borne pathogens

**DOI:** 10.1186/s13071-021-04586-9

**Published:** 2021-02-05

**Authors:** Jörg Hirzmann, David Ebmer, Guillermo J. Sánchez-Contreras, Ana Rubio-García, Gerd Magdowski, Ulrich Gärtner, Anja Taubert, Carlos Hermosilla

**Affiliations:** 1 Institute of Parasitology, Biomedical Research Center Seltersberg, Justus Liebig University Giessen, Schubertstr. 81, 35392 Giessen, Germany; 2 Sealcentre Pieterburen, Hoofdstraat 94a, 9968 AG Pieterburen, The Netherlands; 3 Institute of Anatomy and Cell Biology, Justus Liebig University Giessen, Aulweg 123, 35385 Giessen, Germany

**Keywords:** Pinnipeds, Phocidae, Marine mammal parasitology, Anoplura, Echinophthiriidae

## Abstract

**Background:**

Belonging to the anopluran family Echinophthiriidae, *Echinophthirius horridus*, the seal louse, has been reported to parasitise a broad range of representatives of phocid seals. So far, only a few studies have focused on the vector function of echinophthiriid lice, and knowledge about their role in pathogen transmission is still scarce. The current study aims to investigate the possible vector role of *E.* *horridus* parasitising seals in the Dutch Wadden Sea.

**Methods:**

*E. horridus* seal lice were collected from 54 harbour seals (*Phoca vitulina*) and one grey seal (*Halichoerus grypus*) during their rehabilitation period at the Sealcentre Pieterburen, The Netherlands. DNA was extracted from pooled seal lice of individual seals for molecular detection of the seal heartworm *Acanthocheilonema spirocauda*, the rickettsial intracellular bacterium *Anaplasma phagocytophilum*, and the cell wall-less bacteria *Mycoplasma *spp. using PCR assays.

**Results:**

Seal lice from 35% of the harbour seals (19/54) and from the grey seal proved positive for *A.* *spirocauda*. The seal heartworm was molecularly characterised and phylogenetically analysed (rDNA, *cox1*). A nested PCR was developed for the *cox1* gene to detect *A. spirocauda* stages in seal lice. *A.* *phagocytophilum* and a *Mycoplasma* species previously identified from a patient with disseminated ‘seal finger’ mycoplasmosis were detected for the first time, to our knowledge, in seal lice.

**Conclusions:**

Our findings support the potential vector role of seal lice in the transmission of *A. spirocauda* and reveal new insights into the spectrum of pathogens occurring in seal lice. Studies on vector competence of *E. horridus*, especially for bacterial pathogens, are essentially needed in the future as these pathogens might have detrimental effects on the health of seal populations. Furthermore, studies on the vector role of different echinophthiriid species infecting a wide range of pinniped hosts should be conducted to extend the knowledge of vector-borne pathogens. 
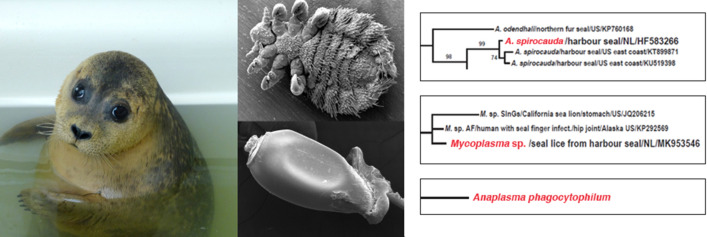

## Background

Representatives of the family Echinophthiriidae belong to the phthirapteran suborder Anoplura, the sucking lice, and exhibit a host range solely infesting semiaquatic mammals, such as pinnipeds (Otariidae, Phocidae, Odobenidae) and the North American river otter (*Lontra canadensis*) [1]. Within the Echinophthiriidae, five genera (*Antarctophthirus, Lepidophthirus*, *Echinophthirius*, *Proechinophthirus*, and *Latagophthirus*) and 13 species are described [1, 2]. While many echinophthiriid species display a strict major host specificity, the seal louse *Echinophthirius horridus* exhibits the broadest host range among echinophthiriid lice parasitising eight different species of Phocidae, the earless or true seals, with a geographical distribution confined to the Northern Hemisphere, including harbour seals (*Phoca vitulina*) and grey seals (*Halichoerus grypus*) [1].

Due to their obligate and permanent hematophagous feeding habits [1], members of the Echinophthiriidae have the potential to play an important role in the epidemiology of vector-borne diseases in free-ranging pinniped populations. Nonetheless, studies on the possible vector function of echinophthiriid species are scarce, but some of them already highlighted their role in potential pathogen transmission (Table 1). The bacterium *Salmonella enteritidis* was isolated from blood and tissue of Northern fur seals (*Callorhinus ursinus*) and from the echinophthiriid lice *Antarctophthirus callorhini* and/or *Proechinophthirus fluctus* collected from the same individuals. Additionally, these infections were also associated with a mortality event of *C. ursinus* pups [3]. A *Rickettsia* species closely related to the human pathogen *Rickettsia* *rickettsi* was isolated from *P.* *fluctus* collected from Northern fur seals [4]. *Bartonella henselae* was detected in *E.* *horridus* and spleen samples of dissected harbour seals [5]. *Southern elephant seal virus* (SESV), an *Alphavirus*, was isolated from *Lepidophthirus macrorhini* parasitising the Southern elephant seal (*Mirounga leonina*) [6].

**Table 1:** Pathogens detected in Echinophthiriidae

Echinophthiriid species	Detected pathogen	References
*Echinophthirius horridus*	*Acanthocheilonema spirocauda*	[7–10, Present study]
	*Bartonella henselae*	[5]
	*Anaplasma phagocytophilum*	[Present study]
	*Mycoplasma* sp.	[Present study]
*Antarctophthirus callorhini*	*Salmonella enteritidis*	[3]
*Proechinophthirus fluctus*	*Salmonella enteritidis*	[3]
	*Rickettsia* sp.	[4]
*Lepidophthirus macrorhini*	*Southern elephant seal virus* (SESV)	[6]

Furthermore, *E.* *horridus* has been suggested to serve as the obligate intermediate host for the seal heartworm *Acanthocheilonema spirocauda*, i.e. *A.* *spirocauda-*microfilariae (L1), taken up by a bloodmeal of the louse, develop into infective L3-stages, which are inoculated to seals during the blood meals [7–9]. The proposed role of *E*. *horridus* as an intermediate host for *A.* *spirocauda* was supported by the finding of different developmental stages in dissected lice [7, 9], the significant positive correlation of infestation of seals with *E.* *horridus* and seal infection with *A*. *spirocauda* [11], and molecular analyses [10].

**Fig. 1:**
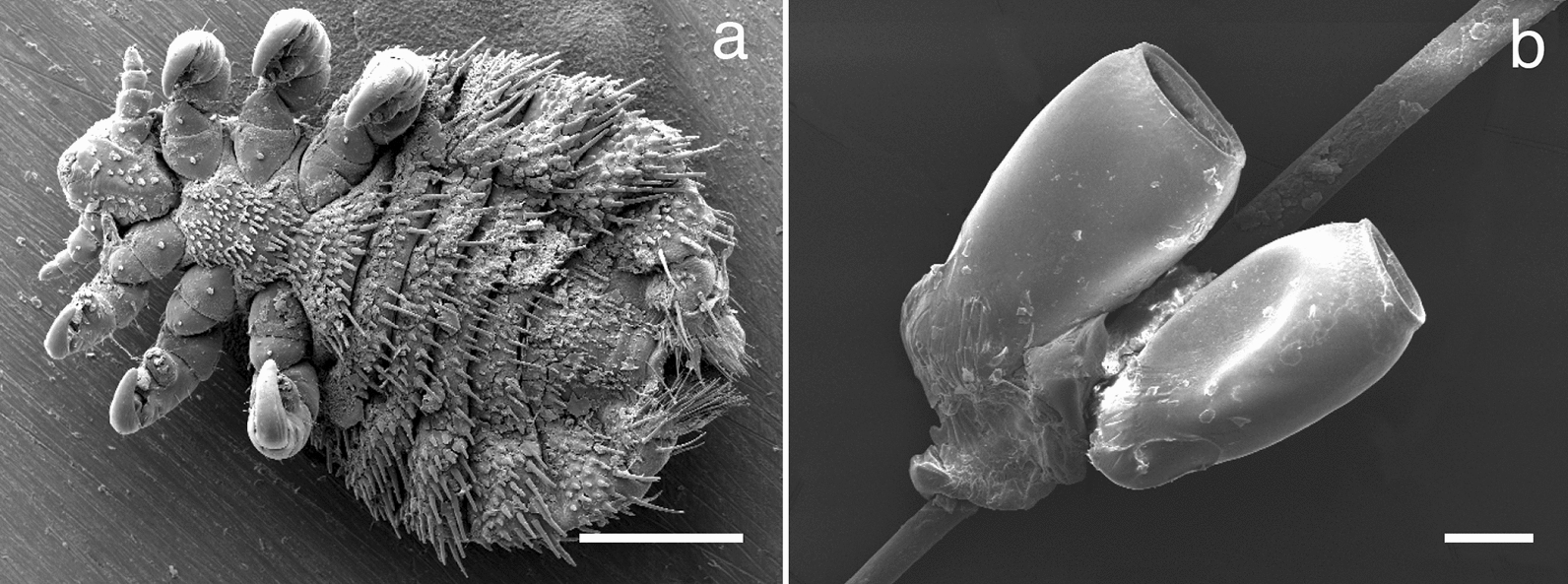
Ultrastructural illustration of *Echinophthirius horridus* adult and egg (nit) stages. (**a**) Ventral view of adult *E. horridus*; (**b**) two opened nits lacking opercula firmly glued to seal fur hair. Scale bars: (**a**) 500 μm, (**h**) 200 μm

Echinophthiriid species exhibit special morphological characteristics, which are unique among anopluran lice and are the basis of their evolutionary success, being influenced by heterogeneous environmental conditions. Thus, distinct spiracles are equipped with special occlusions allowing for gas exchange [12]. Abdominal segments are characterised by a general loss of sclerotization and by a distinct trimming with modified setae [12, 13], sensorial organs, that are classified into spines, scales, and hairs [14]. Furthermore, the last two pairs of legs for fixation to the host surface are another characteristic of echinophthiriid lice. *E.* *horridus* differs from other members of the family by the restructuring of all three pairs of legs into claws and the absence of abdominal scale manifestation (Fig. 1) [14–17].

Due to these morphological adaptions and a strict specifity to semiaquatic host species, a coevolution of echinophthiriid lice and their hosts has been suggested [13, 18, 19]. Thereby, it has been proposed that the ancestors of pinnipeds must have already been infested with ancestral sucking lice before they ventured into the marine habitat [8, 20]. As a consequence of their growing specialisation to ancestral pinnipeds, ancestors of *E. horridus* lice might have evolved a vector role in the transmission of ancestors of the heartworm *A. spirocauda*, which is also believed to have undergone a coevolution with its host species [8]*.* Although filarial infections are usually transmitted by mosquitoes and ticks [21], this filarial heartworm nematode is believed to complete its life cycle in the seal louse *E. horridus* [7]. Nevertheless, it cannot be excluded that other intermediate hosts are involved in transmission of *A. spirocauda*, although suitable mosquito or tick vectors parasitising marine mammals were not reported.

Therefore, the present study aimed to investigate the possible role of *E*. *horridus* in transmission of pathogens, revealing new insights into the spectrum of potential vector-borne diseases circulating in free-ranging seals in the Dutch Wadden Sea.

## Methods

### Sample collection

During routine diagnostics at the Sealcentre Pieterburen (The Netherlands), sucking lice were sampled from 54 hospitalised harbour seals and from one grey seal admitted along the coast of the Dutch Wadden Sea (between 51°42′6.8″N, 3°40′42.1″ E and 53°27′57.6″N, 5°37′40.8″ E) between 6 May and 16 August 2012. Thereby, lice of harbour seals were collected opportunistically using lice combs and forceps and seals were not inspected thoroughly to minimise these potential stress factors. Care was taken to ensure that handling times were reduced to a minimum. If the lice infestation was severe, seals were treated with selamectin (topical) or ivermectin (subcutaneous). Regarding the age structure of the harbour seals, two animals were estimated at around 1 year of age, and all other seals were classified as weaners (< 6 months), including eight pups (< 4 weeks). Thirty of 54 harbour seals were males and 24 females. The grey seal examined in this study died despite intensive medical care during its rehabilitation period. In this case, lice were collected at necropsy.

Collected seal lice specimens as well as eggs (nits) from seal fur, mainly from the head and neck, were fixed in 70% ethanol and morphologically determined by light microscopy. All seal lice stages examined here were classified according to morphological features [1, 14–17]. All fixed specimens were kept at room temperature and delivered to the Institute of Parasitology, Justus Liebig University Giessen, Germany, for further processing. The estimation of the 95% CI was conducted using Kohn and Senyak [22].

### PCR analyses

Seal lice were grouped from each seal and collection date separately, resulting in 64 pools from the 54 harbour seals (*P. vitulina*) containing 1–20 lice and one pool of > 1000 lice from the highly infested grey seal pup divided into pools of 15 lice (Additional file 1). DNA was extracted separately from each seal lice pool. Entire seal lice were washed in distilled water overnight and sliced to minute pieces with sterile scalpel blades in 100 µl of phosphate-buffered saline. DNA was extracted using the DNeasy Blood & Tissue kit (Qiagen) according to the manufacturer’s instructions. PCR assays were performed to detect *A.* *phagocytophilum*, *A.* *spirocauda*, and *Mycoplasma* spp. DNA using as template 500 ng DNA extracted from the lice. For *A.* *phagocytophilum* detection, a partial gene sequence of the *msp2* gene was amplified by real-time PCR according to Courtney et al. [23]. *Mycoplasma* was investigated by conventional PCR amplifying a partial 16S ribosomal DNA sequence with primers MGS0 and GP03 [24]. From the filarial species *A.* *spirocauda*, no sequences were available in NCBI GenBank at the time of this study (GenBank release 193, 12/2012). In a first attempt to obtain sequence data we used pan-filarial primers COIintF/COIintR [25] of the mitochondrial cytochrome c oxidase subunit 1 gene (*cox1*). The obtained sequences from five seal lice pools differed by only one nucleotide position and had a sequence identity of 90% to an *Acanthocheilonema viteae cox1*-sequence (HQ186249) in the GenBank database. The *cox1* sequence from one seal lice pool was submitted to GenBank (accession number HG005138). To validate the sequences from the lice pools and to obtain further sequences from *A. spirocauda,* we then extracted DNA from morphologically confirmed *A.* *spirocauda* adult nematodes (identified according to Leidenberger and Boström [26]; please see Additional file 2), which were collected from necropsied harbour seals at Sealcentre Pieterburen between 2009-2011. Furthermore, we amplified, sequenced, and assembled a partial sequence of the ribosomal DNA region (ITS1, 5.8S, ITS2, and partial 28S) using primers NC5/NC2 [27] and the aforementioned *cox1*-primers.

PCR amplification products were separated on a 2% agarose gel and visualised using Midori Green. Sequencing was performed by an external service provider (LGC Genomics, Berlin, Germany) and the new sequences were deposited in GenBank database (accession numbers: *A.* *spirocauda*: HF583266, *Mycoplasma* sp.: MK953546). The *cox1* amplicon sequence from the adult *A. spirocauda* (accession number HF583266) had an additional single-nucleotide polymorphism (not shown) compared to the sequences obtained from the DNA extracted from pools of infested lice.

For the molecular detection of *A. spirocauda* larvae in seal lice a more sensitive nested PCR (outer primers COIintF/COIintR; inner primers AspF/AspR) was established based on the alignment of *cox1* sequences from *A.* *spirocauda*, *A.* *viteae* and *Dirofilaria immitis*. Primers AspF (5'-TGCTGTTACTTTGGACCAGGT-3') and AspR (5'-ATGATGGCCCCACACAGAAG-3') were designed with Primer3 software [28]. The first PCR (COIintF/COIintR) was performed in a reaction volume of 50 µl containing 5 µl template DNA, 5 µl 10× PCR buffer S (Peqlab, VWR International GmbH, Erlangen, Germany), 1 µl of each primer, 1 µl dNTPs, 1 µl 5 U/µl peqGOLD “Hot” Taq DNA Polymerase (Peqlab, VWR International GmbH, Erlangen, Germany), and 36 µl H_2_0. The following conditions were used: 2 min 95 °C initial denaturation, 35 cycles of 30 s 94 °C, 30 s annealing at 50 °C, 45 s 72 °C, and 5 min 72 °C final extension. For the second PCR (AspF/AspR), 1 µl of the first PCR served as template in a 50 µl reaction containing 5 µl 10× PCR buffer S, 1 µl of each primer, 1 µl dNTPs, 1 µl peqGOLD “Hot” Taq DNA Polymerase, and 40 µl H_2_0, under the following conditions: 2 min 95 °C initial denaturation, 35 cycles of 30 s 94 °C, 30 s annealing at 58 °C, 30 s 72 °C, and 5 min 72 °C final extension. PCR products were separated on a 2% agarose gel stained with Midori Green. The achieved sensitivity of this method was determined using the counted number of microfilariae (see Additional file 3) isolated from gravid *A.* *spirocauda*.

### Phylogenetic analyses

For molecular phylogenetic analyses data sets of highly matching sequences to *A.* *spirocauda* and *Mycoplasma* sp. sequences were obtained from BLAST searches of the GenBank database (GenBank release 238, 06/2020); sequences were trimmed to homologous ends and realigned using the multiple sequence alignment program MAFFT 7 [29] with the L-INS-i method for the *cox1* and 16S sequence data sets and the structure-aided Q-INS-i method for the ITS2 sequence data set. Phylogenetic trees were constructed using Bayesian analysis (MrBayes 3.2) (10.000 tree generations, sampling each 10, discarding first 250 trees) and TreeDyn for tree drawing at the phylogeny.fr platform [30]. The data sets included sequences homologous to nucleotides (nt) 122–624 of the *A.* *spirocauda cox1* sequence (HF583266K), nt 466–942 of the *A.* *spirocauda* ITS2 sequence (HG005138), and nt 1–217 of the *Mycoplasma* sp. 16S ribosomal RNA gene sequence (MK953546). The *A.* *spirocauda cox1* sequence of an isolate from *Erignathus barbatus* (bearded seal) in Barrow (Alaska, USA) (KF038155) [31] was not included because the overlapping sequence to the other *A.* *spirocauda cox1* sequences (excluding KT899872) was too short (169 nt, intraspecies variation 2.4%, i.e. 4/169 nt).

## Results

### Seal lice and vector-borne pathogens

In total, 200 adult *E.* *horridus* seal lice were collected separately from 54 harbour seals (infestation 1–20 lice/seal, median 2; see Additional file 1) and more than 1000 lice from a single grey seal (*H.* *grypus*) pup. Seal lice from 19 of the 54 infested harbour seals (prevalence 35.2%; 95% CI 22.5–47.9%) and from the highly infested grey seal pup proved positive for *A.* *spirocauda*. In addition, seal lice (8 pools of 15 specimens) from the grey seal were found positive for *A.* *phagocytophilum* and seal lice from three harbour seals were found positive for *Mycoplasma* sp. Analysis of the partial 16S sequence revealed that a single strain of *Mycoplasma* sp. was present in the seal lice obtained from three harbour seals, which was identical to a *Mycoplasma* sp. from an Alaska native hunter who suffered from disseminated seal finger mycoplasmosis [32].

In an intial *cox1*-PCR using pan-filarial primers and sequencing the obtained sequences from lice pools and morphological confirmed *A.* *spirocauda* adults differed by only 3/649 nt (intraspecies variation 0.5%). We compared the *cox1* single and nested PCR on ten pools. The nested PCR was more sensitive for these pools and therefore we decided to apply the nested PCR as diagnostic PCR for all pools. It had a consistent minimal detection of ten microfilariae (see Additional file 3) and was sufficient to detect *A. spirocauda* larvae in a single louse (seven positive lice pools from the harbour seals contained only one seal louse; see table in Additional file 1).

### Phylogenetic analysis of *A. spirocauda* and the *Mycoplasma* isolate

**Fig. 2:**
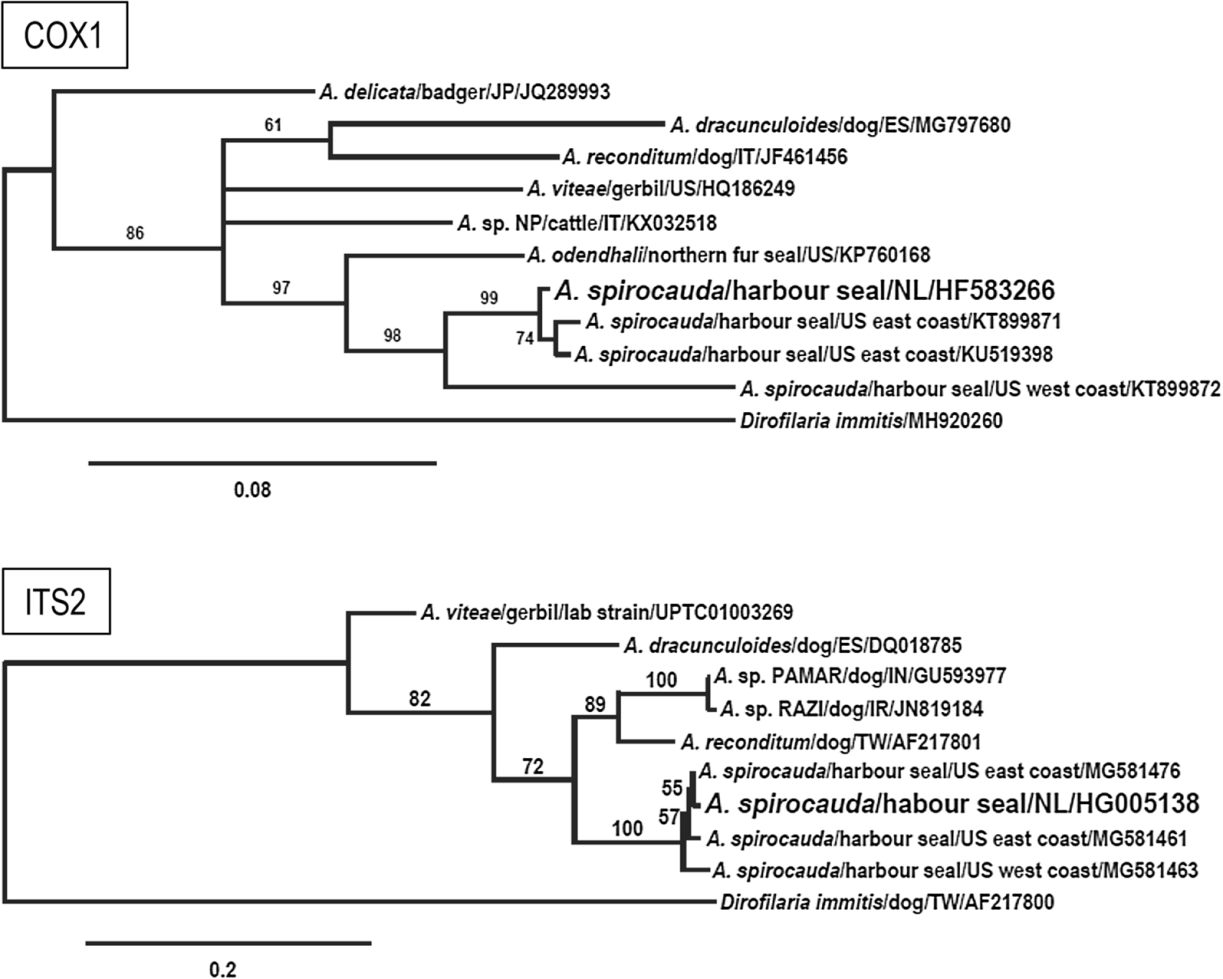
Phylogenetic analysis of *A.* *spirocauda*. The phylogeny is based on the MAFFT alignments for a partial *cox1* sequence and complete ITS2 rDNA region of *A.* *spirocauda* and other *Acanthocheilonema* species using Bayesian inference (*Dirofilaria immitis* sequence as outgroup). The *A.* *dracunculoides* ITS2 sequence is shorter and does not cover the whole sequence of *A*. *spirocauda*. Branch lengths are drawn proportionally to evolutionary distance (scale bar is shown). Numbers adjacent to nodes indicate posterior probabilities in per cent. Branch labels provide species names/host/two-letter country code/GenBank accession number

**Fig. 3:**
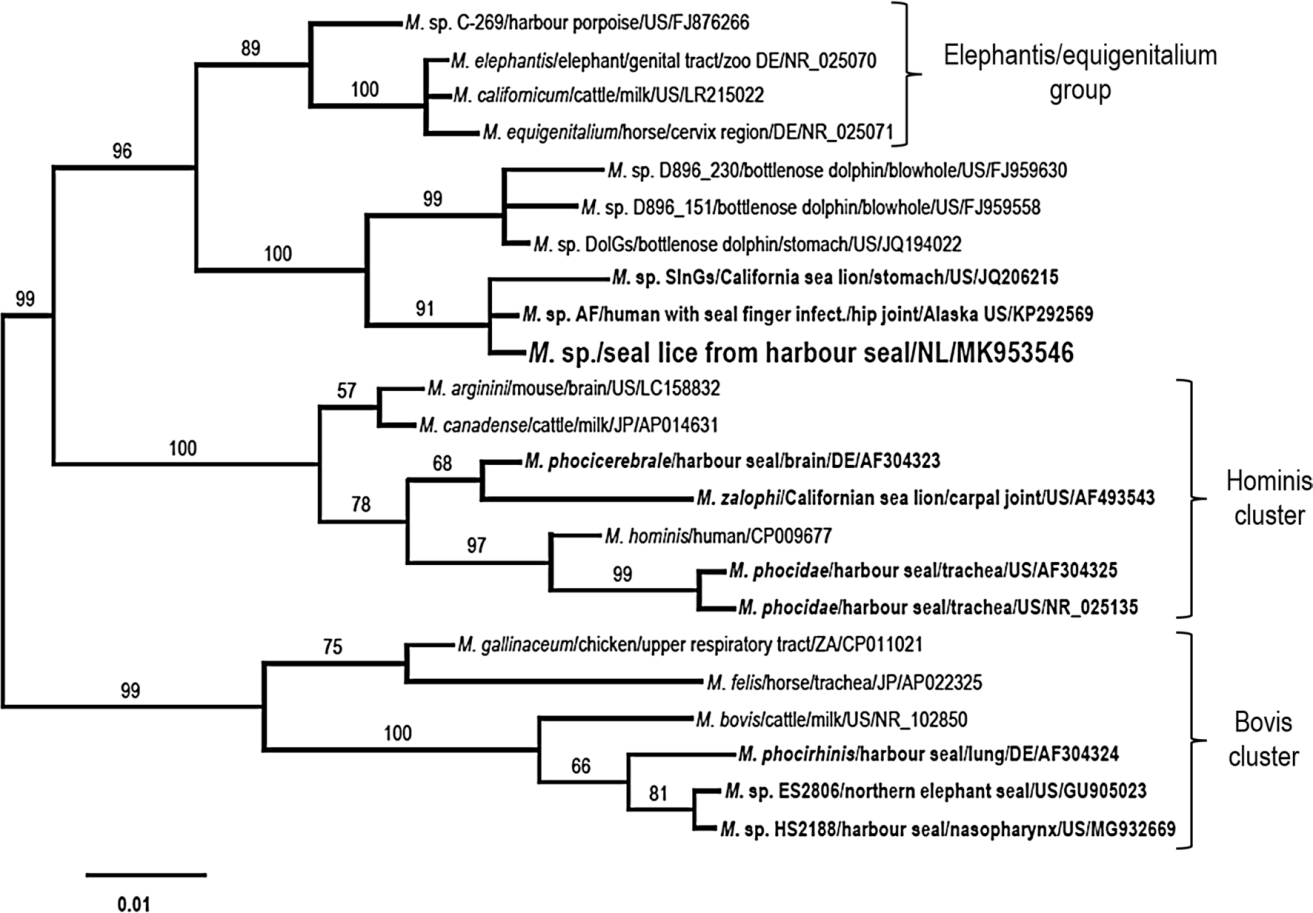
Phylogenetic analysis of *Mycoplasma* sp. from seal lice collected from harbour seals. Bayesian phylogenetic tree of *Mycoplasma* sp. of seal lice from harbour seals of the present study and phylogenetically closely related *Mycoplasma* species based on partial 16S gene sequences. Branch lengths are drawn proportionally to evolutionary distance (scale bar is shown). Numbers adjacent to nodes indicate posterior probabilities in per cent. Branch labels provide species names/host/source/two-letter country code/GenBank accession number. *Mycoplasma* species from seals highlighted in bold

Phylogenetic analysis of the partial *cox1* sequence showed that *A. spirocauda* was most closely related to *A. odendhali* (Fig. 2), a filarial parasite of the Northern fur seal and the California sea lion (*Zalophus californianus*) occurring in subcutaneous and intermuscular sites and considered to be non-pathogenic [31, 33]. Interestingly, one *A.* *spirocauda* isolate (PPr11-007; accession numbers *cox1* KT899872, ITS2 MG581463) collected from a stranded, deceased harbour seal from the USA west coast (Stinson Beach, CA) [10] differs from all other *A.* *spirocauda* isolates by some indels in the ITS2 region and point mutations in the partial *cox1* gene resulting in only 95% and 93% sequence identity respectively (not shown). The partial 16S sequence of the *Mycoplasma* species detected in seal lice of three harbour seals in this study was identical to the sequence from an infected human patient (GenBank accession no. KP292569). This zoonotic *Mycoplasma* species forms a new phylogenetic group together with *Mycoplasma* sp. isolates from common bottlenose dolphins (*Tursiops truncatus*) and from a California sea lion (Fig. 3). It is positioned next to the elephantis-equigenitalium group and clearly separated from other *Mycoplasma* species previously described infecting pinnipeds, such as *M.* *phocicerebrale, M. phocidae*, *M.* *phocirhinis* and *M.* *zalophi*.

### Severe *E. horridus* infestation in a grey seal

Investigation of a grey seal which was found stranded on the beach revealed a severe *E. horridus* infestation (> 1000 specimens). The animal weighted 15 kg at admission to the Sealcentre Pieterburen and was estimated on its teeth development as a few week old pup. On arrival to the Sealcentre Pieterburen the male pup was cachectic, showing fever and severe dehydration. After 14 days of intensive medical care treatments, the pup did not improve and died.

At necropsy, gross pathology findings included consolidation of large portions of the lungs and presence of foam and fluids in the trachea and main bronchial trees. Histopathological examination of the lungs revealed acute interstitial pneumonia, which was diagnosed as the cause of animal death. No *A. spirocauda* infection was detected during necropsy. Unfortunately, no blood samples were left from this grey seal in order to perform blood smears and additional diagnostic PCRs to determine whether the grey seal was positive for *A.* *phagocytophilum* too.

## Discussion

This study documents the presence of helminth and bacterial pathogens in the seal louse *E. horridus*, corroborating that this arthropod is a vector of *A. spirocauda* and suggesting that it may also play a role in the epidemiology of *A. phagocytophilum* and seal finger-associated *Mycoplasma* sp. The presence of *A. spirocauda* larvae in *E. horridus* was already described in the past [7–9, 10] and molecularly confirmed in the present study. Our results showing that seal lice from 35% of the harbour seals and from the grey seal proved positive for *A.* *spirocauda* strongly support the hypothesis that *E. horridus* functions as obligate intermediate host of *A. spirocauda*. Furthermore, *A. spirocauda,* the only filarial nematode parasite of phocid seals [8, 34], was molecularly characterised in this study.

Regarding the epidemiology and pathogenicity of echinophthiriosis, it is more frequently reported in young and weak animals [7, 8, 35], showing no seasonal variations for adult seals, but pups and immature seals have higher prevalences in spring. In contrast, Dailey and Fallace [11] reported the highest prevalence in the autumn and winter months, but no significant differences between examined age classes of seals and their seal lice burden were detected. Interestingly, in the closely related species *A.* *microchir* from the South American sea lion over 60% of 1-day-old pups were infested with lice, and recruitment increased in pups up to 3 days old and leveled off onwards. In 1-day-old pups, significantly more adults than nymphs were found, but this pattern was reversed in older pups, documenting the importance of vertical transmission probably through their mothers [36].

Molecular analyses on collected seal lice from harbour and grey seals revealed the presence of DNA of the seal heartworm *A.* *spirocauda*. In this context, *E.* *horridus* has previously been proposed to be the natural obligate intermediate host of *A.* *spirocauda* [7–9, 10], and different stages of *A.* *spirocauda* larvae were found in dissected *E.* *horridus* seal lice [7, 9]. So far, the heartworm *A.* *spirocauda* has been reported from different phocid species such as harbour seals, hooded seals (*Cystophora* *cristata)*, bearded seals, ribbon seals (*Phoca* *fasciata*), harp seals (*Phoca* *groenlandica*), ringed seals, spotted seals (*Phoca* *largha*), monk seals (*Monachus* *monachus*), and recently from grey seals [8, 10, 37]. Furthermore, there is a significant positive correlation between heartworm infection and infestation of harbour seals with seal lice [11]. The nested PCR protocol developed in this study had a detection threshold as low as ten microfilariae and therefore may be a useful method for future studies on the epidemiology of these parasites in seal lice. Recently, Keroack et al. [10] developed a more sensitive *A.* *spirocauda* real-time quantitative PCR based on a highly repetitive genomic DNA repeat identified using whole-genome sequencing that could also improve future monitoring of seal heartworm infections. These authors also identified the first time an *A.* *spirocauda* adult worm in a presumed grey seal carcass from the coast of Cape Cod (MA, USA). However, the authors mentioned that the carcass was in very poor condition due to extensive decomposition and could not be fully identified and reported just the evidence for possible *A. spirocauda* infection in the grey seal.

Moreover, to our knowledge our results represent the first detection of *A.* *phagocytophilum* and *Mycoplasma* sp. in seal lice. However, based on the limited knowledge of vector-borne pathogens occurring in marine habitats, our findings of *A. phagocytophilum* and *Mycoplasma* sp. do not necessarily imply that the seal louse is a viable vector of these pathogens. Analogous to studies on the vector competence of human body lice [38, 39], evidence of vector competence of *E. horridus* and the ability of transmitting these pathogens could only be proved by experimental infections. Using molecular methods, it cannot be excluded that the pathogens detected were located in the gastrointestinal tract of *E. horridus* individuals after blood consumption of infected seals but are not able to be transmitted to other host individuals. Nevertheless, molecular surveys of the present study constitute important baseline studies in the field of marine mammal parasitology to initially reveal a spectrum of pathogens, which could possibly be transmitted by seal lice. Thereby, our results can also help to encourage other researchers to extend the knowledge of vector-borne pathogens in the field of marine mammal parasitology.

To our knowledge, there are no reports on *A.* *phagocytophilum* or *Mycoplasma* spp. in any other ectoparasite affecting marine mammals or evidence of anaplasmosis occurring in stranded phocid seals [40, 41]. *Anaplasma phagocytophilum* (Rickettsiales, Anaplasmataceae) constitutes an emerging globally distributed pathogen transmitted mainly by *Ixodes* ticks and may cause granulocytic anaplasmosis, one of the most relevant tick-borne diseases of veterinary and public health significance worldwide [42]. Virtually nothing is known about the occurrence and clinical implications of *A. phagocytophilum* in seals, and the detection of this pathogen in *E. horridus* is surprising since to our knowledge there are no records of tick parasitism on pinnipeds. This raises the question of whether other haematophagous ectoparasites (such as *E. horridus*) might play a role in the transmission of *A. phagocytophilum* among marine mammals.

Variable clinical signs of granulocytic anaplasmosis can include high fever, lethargy, inappetence, anorexia, dullness, reduced weight gain, coughing, and abortion in different animal species in Europe, including domestic ruminants, horses, dogs, and cats [42–44]. In humans, symptoms were reported as non-specific and include influenza-similar symptoms with fever and myalgia. In addition, leucopenia, thrombocytopenia and/or anaemia have been frequently reported to occur in certain *A.* *phagocytophilum* strain infections [43]. Many of these non-specific clinical signs were observed in the heavily *E. horridus*-infested grey seal pup evaluated in this study, for which ~ 50% of the seal louse pooled samples were positive for *A. phagocytophilum*, suggesting that this individual might have suffered from acute granulocytic anaplasmosis. The zoonotic potential of the *A. phagocytophilum* genetic variant detected here cannot be assessed by the *msp2* sequence and needs further characterisation. In this context, it would also be interesting to investigate whether red foxes (*Vulpes vulpes*)—frequent hosts of *A. phagocytophilum*—living in the Dutch coastal dune area might occasionally feed on dead seal pups and get infested by *E*. *horridus* as has been reported for Arctic foxes (*Vulpes lagopus*) from Alaska and the fur seal louse (*Antarctophthirus callorhini*) [1]. In this context, it is worth noting that red fox activity was observed in the UK within a mainland grey seal breeding colony [45] and that satellite tracking studies have shown that grey seals captured in The Netherlands may migrate to breed in the UK [46].

*Mycoplasma* sp. was also molecularly identified in collected seal lice specimens in the current study, and in contrast to *A.* *phagocytophilum* there are previous reports on the occurrence of mycoplasmal infections in pinnipeds [47–52]. *Mycoplasma* spp. are common inhabitants of the respiratory, gastrointestinal and genital tracts of marine mammals, and a study on Australian fur seals (*Arctocephalus pusillus doriferus*) demonstrated the presence of different *Mycoplasma* species such as *M.* *phocicerebrale*, *M.* *phocidae*, *M.* *zalophi,* and *Mycoplasma* sp. in tested animals [49]. Furthermore, PCR testing of nasal swabs detected the presence of *Mycoplasma* spp. DNA in South American fur seal (*Arctocephalus australis*) populations in Peru with an estimated prevalence of around 38% [53], evidencing rather cosmopolitan distribution of mycoplasmas in pinnipeds. For universal detection of mycoplasmas the highly specific and sensitive PCR assay of van Kuppeveld [24] was used in the current study based on the conserved region of the 16S gene. Direct sequencing and sequencing of several clones indicated a single strain only. However, better species differentiation would be possible selecting further housekeeping genes (e.g. *rpoB*, *rpoC*) and culturing for phenotypical characterisation and serological testing. Therefore, our findings on *Mycoplasma*-positive *E.* *horridus* lice might suggest the presence of these bacterial infections in pinnipeds of the Dutch Wadden Sea. Whether seal lice might be potentially involved in the transmission of *Mycoplasma* will require further investigation.

Nevertheless, it is significant to note that some mycoplasmas (e.g. *M. phocicerebrale*) are associated with seal mortality and zoonotic ‘seal-finger’ infection, a disease known among people who handle seals [54]. Seal-finger lesions could progress to septic arthritis of the joints if tetracycline-based treatment is not received. Accordingly, more recent published studies on a series of bites and contact abrasion in open-water swimmers caused by California sea lions and harbour seals revealed the presence of *Mycoplasma* spp. in human wounds [52, 55], demonstrating its zoonotic potential. GenBank database search using the partial *Mycoplasma* sp. 16S sequence, detected in seal lice collected from three harbour seals in the current study resulted in 100% identity to the sequence of an unnamed *Mycoplasma* strain (GenBank accession number KP292569) obtained from a patient with “seal finger” and an infected hip joint. The patient previously hunted and harvested ringed seals (*Phoca hispida*) in Alaska without protective gloves in an area where *Mycoplasma*-infected seals had been noticed before [32].

## Conclusions

In conclusion, this study shows the occurrence of potential pathogens within seal lice of infested pinnipeds in the Dutch Wadden Sea and reveals new insights into the potential role of *E. horridus* as vector. Thereby, the current study molecularly confirms former suggestions that E. horridus functions as an obligate intermediate host for the seal heartworm *A. spirocauda*. Whether *E. horridus* plays a role in the transmission of granulocytic anaplasmosis and *Mycoplasma* spp. infections in seals remains to be clarified by future studies.

## Supplementary Information

**Additional file 1.** Infestation of individual seals with seal lice.

**Additional file 2.** Morphological features of *A. spirocauda*.

**Additional file 3.** Sensitivity of the *Acanthocheilonema spirocauda cox1* nested-PCR.

## Data Availability

All data generated or analysed during this study are included in this published article and its supplementary information files.
