# Supplementary material for: The seal louse (Echinophthirius horridus) in the Dutch Wadden Sea: investigation of vector-borne pathogens
Source: Parasit Vectors. 2021 Feb 5;14:96. doi: 10.1186/s13071-021-04586-9 (PMC7863525; doi:10.1186/s13071-021-04586-9)
Supplement: Supplementary file 1 — Additional file 1. Infestation of individual seals with seal lice. [file 13071_2021_4586_MOESM1_ESM.pdf]

Additional file 1
Infestation of seals with seal lice (after arrival at Sealcentre Pieterburen)

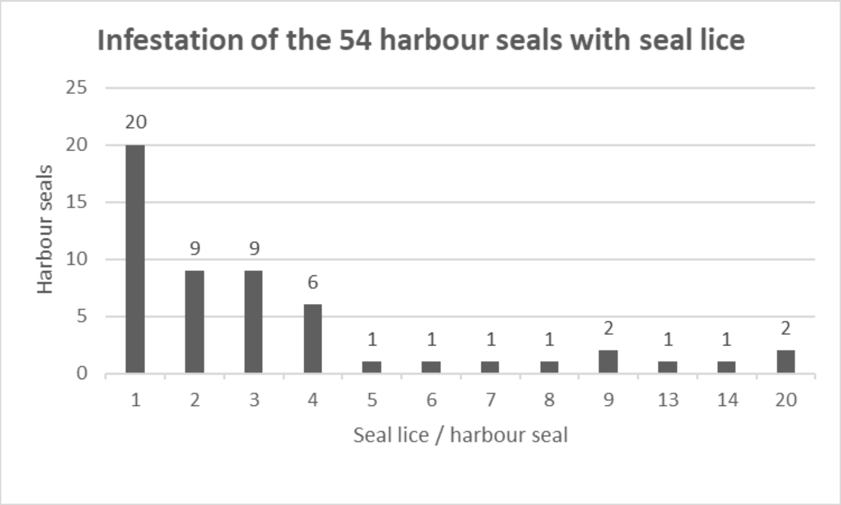

Number of *Echinophthirius horridus* lice detected on seals admitted to Sealcentre Pieterburen (Pieterburen, The Netherlands) for rehabilitation in summer 2012 (05-06 – 08-16-2012). Lice were pooled separately from each seal and were molecularly analyzed for pathogens.

Pool no.	Number of lice	Rehab number	Species	Collection date
—	>1000	**12-289**	HG	5-6-2012
—	8 mites	"	HG	5-6-2012
1	3	12-307	PV	6-2-2012
2	1	12-312	PV	6-17-2012
**3**	1	12-318	PV	6-11-2012
4	1	12-321	PV	8-16-2012
5	9	12-322	PV	7-14-2012
6	4	"	PV	7-16-2012
7	1	12-340	PV	6-17-2012
8	1	12-344	PV	8-11-2012
9	2	12-351	PV	7-5-2012
**10**	1	**12-354**	PV	6-19-2012
11	1	12-356	PV	6-30-2012
12	2	"	PV	7-4-2012
13	2	12-357	PV	7-23-2012
14	2	12-358	PV	6-21-2012
15	2	12-359	PV	7-5-2012
16	1	12-360	PV	7-23-2012
17	4	12-372	PV	7-11-2012
18	2	12-375	PV	6-27-2012
19	2	"	PV	7-9-2012
20	3	12-378	PV	6-27-2012
21	3	12-381	PV	6-30-2012
**22**	4	**12-385**	PV	6-26-2012
23	16	"	PV	7-11-2012
**24**	2	**12-386**	PV	7-3-2012
25	6	"	PV	7-15-2012
**26**	1	**12-389**	PV	6-26-2012
27	2	12-390	PV	7-2-2012
**28**	1	**12-391**	PV	6-27-2012
29	2	12-393	PV	7-29-2012
30	5	12-397	PV	7-11-2012
31	3	12-399	PV	7-2-2012

**Pool no.**	**Number of lice**	**Rehab number**	**Species**	**Collection date**
32	1	12-400	PV	7-3-2012
33	1	12-403	PV	7-4-2012
**34**	3	**12-404**	PV	7-4-2012
35	3	"	PV	8-8-2012
**36**	2	**12-406**	PV	7-4-2012
37	1	"	PV	7-13-2012
38	1	"	PV	?
39	1	12-408	PV	7-4-2012
40	1	12-409	PV	7-28-2012
41	3	12-410	PV	7-3-2012
42	2	12-418	PV	11-7-2012
43	1	12-419	PV	8-14-2012
44	1	12-421	PV	8-14-2012
45	4	12-422	PV	8-13-2012
**46**	4	**12-424**	PV	7-19-2012
47	20	12-425	PV	7-19-2012
**48**	1	**12-427**	PV	7-22-2012
49	1	"	PV	8-13-2012
**50**	3	**12-428**	PV	7-21-2012
51	3	12-429	PV	7-24-2012
**52**	2	**12-430**	PV	7-24-2012
53	1	"	PV	8-13-2012
**54**	1	**12-432**	PV	24-7-2012
55	1	12-433	PV	7-27-2012
56	9	12-434	PV	7-29-2012
**57**	4	**12-435**	PV	7-29-2012
**58**	7	**12-436**	PV	8-2-2012
**59**	2	**12-438**	PV	7-30-2012
**60**	1	**12-439**	PV	8-6-2012
**61**	9	**12-443**	PV	8-6-2012
62	1	12-445	PV	?
63	1	12-454	PV	8-15-2012
**64**	14	**120709-06**	PV	7-12-2012

Seals: HG *Halichoerus grypus*, PV *Phoca vitulina*, **pools in bold** were positive for *Acanthocheilonema spirocauda*.
