# Supplementary material for: The seal louse (Echinophthirius horridus) in the Dutch Wadden Sea: investigation of vector-borne pathogens
Source: Parasit Vectors. 2021 Feb 5;14:96. doi: 10.1186/s13071-021-04586-9 (PMC7863525; doi:10.1186/s13071-021-04586-9)
Supplement: Supplementary file 2 — Additional file 2. Morphological features of A. spirocauda. [file 13071_2021_4586_MOESM2_ESM.pdf]

Additional file 2

Morphological features of *Acanthocheilonema spirocauda* collected from necroptized harbour seals at Sealcentre Pieterburen.

Filariae collected from the heart of necropsied habour seals were identified to species level by morphological features and compared to the data from Anderson (1959) and Leidenberger and Boström (2008).

Female

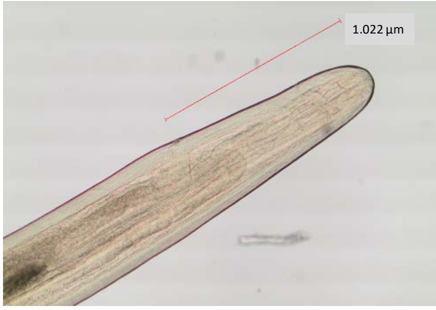

Vulva to anterior end

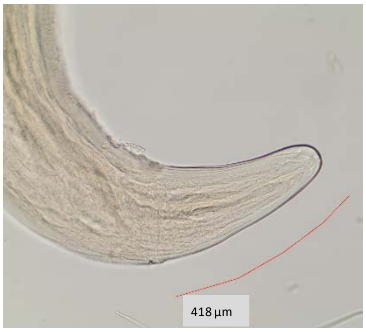

Anus to posterior end

Male

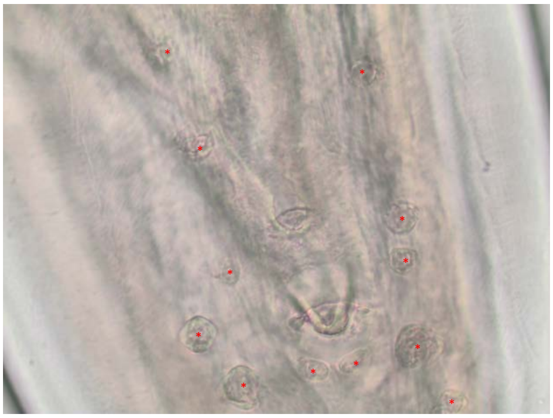

Papillae arround the cloacal opening (*):

- One unpaired and three pairs of preanal papillae
- Four papillae in row immediately behind anus, the outer being larger, the inner oval and small
- one unpaired papilla behind the anus

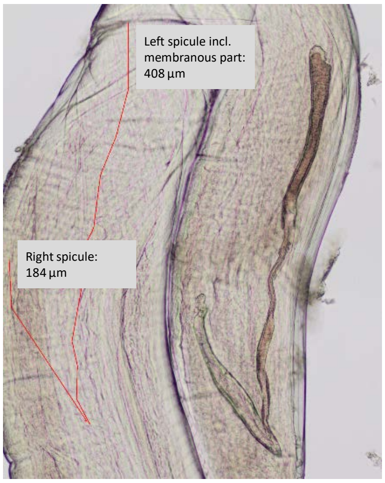

Length of spicules: the left spicule (253 µm) has an extended, chitinized membranous part (sheath).

Length of *Acanthocheilonema spirocauda*
Male: 8.5 – 9.5 cm (mean 9.2 cm, n=3)
Female: 13 – 17.5 cm (mean 15.8 cm, n=6)
Microfilariae from terminal uterus: 186 – 189 µm (mean 188 µm, n=3)

Anderson RC. The taxonomy of Dipetalonema spirocauda (Leidy, 1858) n. comb. (=Skrjabinaria spirocauda) and Dirofilaria roemeri (Linstow, 1905) n. comb. (=Dipetalonema roemeri). Can J Zool. 1959;37:481–493. https://doi.org/10.1139/z59-057

Leidenberger S, Boström S. Characterization of the heartworm Acanthocheilonema spirocauda (Leidy, 1858) Anderson, 1992 (Nematoda: Onchocercidae) in Scandinavia. Parasitol. Res. 2008;104: 63-67. https://doi.org/10.1007/s00436-008-1159-1
