# Supplementary material for: The seal louse (Echinophthirius horridus) in the Dutch Wadden Sea: investigation of vector-borne pathogens
Source: Parasit Vectors. 2021 Feb 5;14:96. doi: 10.1186/s13071-021-04586-9 (PMC7863525; doi:10.1186/s13071-021-04586-9)
Supplement: Supplementary file 3 — Additional file 3. Sensitivity of the Acanthocheilonema spirocauda cox1 nested-PCR. [file 13071_2021_4586_MOESM3_ESM.pdf]

Additional File 3
Sensitivity of the *Acanthocheilonema spirocauda cox1* nested-PCR

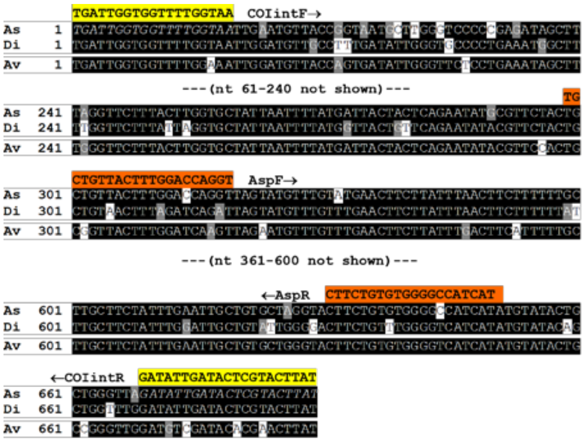

Localisation of diagnostic PCR-primers and alignment of the partial *A. spirocauda cox1*-sequence (As) with sequences of *Dirofilaria immitis* (Di, AJ537512) and *Acanthocheilonema viteae* (Av, HQ186249)

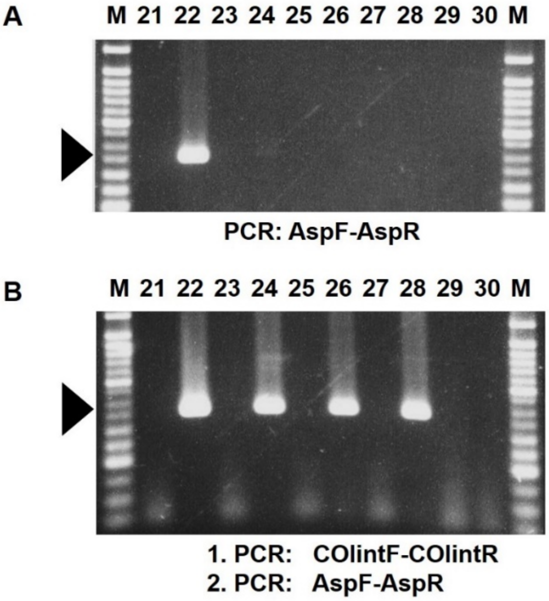

Comparison in sensitivity of the *A. spirocauda cox1* PCR (**A**) and the *cox1* nested-PCR (**B**), lice pools 21-30 from harbour seals, specific 351 bp amplicon (arrow heads), M=molecular weight standard (50 bp ladder)

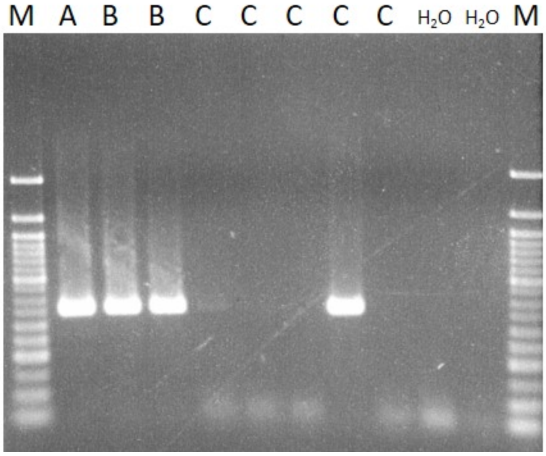

Microfilariae isolated from adult female *A. spirocauda* were resuspended in PBS and counted under lightmicroscopy. Dilutions of 200 mf/10 µl (A), 20 mf/10 µl (B), and 2-4 mf/10 µl (C) were prepared. Each batch was digested with 1 µl of Proteinase K (20 mg/ml) for 2 h at 56 °C and deactivated 5 min at 95 °C. 5 µl were used as template for the first PCR (COIintF/COIintR) in a 50 µl reaction mix (conditions: 2´ 95 °C/ 35x 30´´ 94 °C, 30´´ 50 °C, 45´´ 72 °C/ 5´ 72°C). 1 µl of the first PCR was used as template in a 50 µl nested PCR (Asp-F/Asp-R; 2´95 °C/ 35x 30´´ 94 °C, 30´´ 58 °C, 30´´ 72 °C) and 8 µl were seperated on a 2% agarose gel; M=50 bp molecular weight standard.
